# Quality of life of caregivers of breast cancer patients: a cross-sectional evaluation

**DOI:** 10.1186/s12955-022-01930-0

**Published:** 2022-02-19

**Authors:** Marloes E. Clarijs, Arvind Oemrawsingh, Mirelle E. E. Bröker, Cornelis Verhoef, Hester Lingsma, Linetta B. Koppert

**Affiliations:** 1 Academic Breast Cancer Center, Department of Surgical Oncology and Gastrointestinal Surgery, Erasmus MC Cancer Institute, Erasmus University Medical Center, Room NA-2117, 3000 WB Rotterdam, The Netherlands; 2 Center for Medical Decision Making, Department of Public Health, Erasmus University Medical Center, Rotterdam, The Netherlands

**Keywords:** Breast cancer, Caregivers, Informal care, Quality of life

## Abstract

**Background:**

The aim of this study was to evaluate the care-related quality of life in caregivers of breast cancer patients, to assess its association with breast cancer patients’ health-related quality of life (HRQoL), and to identify its potential predictors.

**Methods:**

Caregivers of breast cancer patients at six and twelve months follow up were identified through the institutes electronic patient reported outcome measurement collection tool. The Care-related Quality of Life Instrument (CarerQoL) was used to obtain CarerQoL utility scores by applying a pre-existent set of Dutch tariffs and the CarerQoL VAS score, which represented the overall happiness of caregivers. The associations between breast cancer patients’ EQ-5D-5L and EORTC QLQ-C30 scores and caregivers’ CarerQoL scores was determined with Spearman’s correlation coefficients. Associations between log transformed CarerQol scores and patient and caregiver characteristics were analyzed with multivariable linear regression analyses.

**Results:**

A total of 116 completed CarerQoL questionnaires were analyzed. Most caregivers were male spouses or partners (81.4%) with a mean age of 55.7 ± 16.4. The median CarerQoL utility score was 92.4/100 and median CarerQoL VAS was 8.0/10. We found weak correlations between CarerQoL VAS scores and patients’ EQ-5D-5L utility score (0.301, *p* = 0.002) and EQ VAS score (0.251, *p* = 0.009), and between EORTC QLQ-C30 scores and CarerQol VAS (0.339, *p* < 0.001) and utility score (0.236, *p* = 0.015). There was a negative association between chemotherapy and log-transformed CarerQoL utility score (B =  − 0.063, *p* = 0.001) and VAS score (B =  − 0.044, *p* = 0.038) at six months follow-up.

**Conclusions:**

This study provides the first evaluation of the CarerQoL in caregivers of Dutch breast cancer patients. Caregivers’ happiness was associated with breast cancer patients’ HRQoL. Our results can be used as reference values for future care-related quality of life evaluations.

**Plain English Summary:**

Breast cancer patients face many difficulties during their cancer journey and often need the support of their caregivers. Despite the fact that successfully providing informal care can have positive effects on caregivers’ wellbeing, it may also have a negative impact on their quality of life. Monitoring the quality of life using a standardized questionnaire, such as the CarerQoL questionnaire, may result in early detection of possible quality of life issues. In this study, we evaluated 116 caregivers and found overall high CarerQoL scores. The scores showed a positive relation to the patients’ quality of life. Lower CarerQoL scores at six months after surgery were found in caregivers of patients who received chemotherapy. Our research underlines the importance to include caregivers of breast cancer patients in clinical practice, provides reference values for future research, and the results can be used to manage the caregivers’ expectations prior to treatment.

## Background

In the Netherlands, approximately 23% of the population provides informal care for various health indications [1]. Survival rates for breast cancer patients have improved over the last years [2]. Informal care plays an essential part during their diagnostic and treatment process. Having a social network ensures access to informal care which may even positively affect breast cancer outcomes [3, 4].

The term ‘informal care’ is often interpreted in the context of chronically ill or severely disabled patients in need of daily support and care. However, there is a wide variation in definitions of informal care and it is provided in many forms and in all kinds of situations. Informal care may include support during medical visits, managing wound or drain care, managing medication intake or other activities of daily living, and is performed voluntary by non-professional people without compensation. In addition to physical care, the social-emotional support of caregivers has a positive effect during decision making and processing [5].

As shortening of hospital-based care and early hospital discharge after breast surgery has been shown to improve clinical outcomes [6, 7], support for at-home recovery is often required. Additionally, systemic therapies (e.g. chemotherapy) are increasingly being offered to women with breast cancer, which may cause uncomfortable side effects requiring care at home. Breast cancer patients face many difficult decisions during their cancer journey. These situations illustrate that the burden of caregivers is growing. Caregivers often feel obliged to provide informal care to their relatives. Despite the fact that successfully providing informal care can have positive effects on caregivers’ wellbeing, it may also have a negative impact on their lives [8]. Stress or anxiety induced by continuous caregiving may result in health issues and indirectly affect the care recipient [9, 10]. Monitoring the care-related quality of life of caregivers by using a standardized questionnaire may result in early detection of possible financial, relational or health problems [11, 12]. Thus, engaging caregivers during the treatment of breast cancer patients and optimizing the communication between provider, patient and caregiver may lead to better patient outcomes and breast cancer care.

The primary aim of the current study was to evaluate the care-related quality of life in caregivers of breast cancer patients using the Care-related Quality of Life Instrument (CarerQoL). The primary outcome was the CarerQoL utility score. The second aim was to correlate the CarerQoL utility and VAS scores with health-related quality of life (HRQoL) scores of breast cancer patients and to identify potential predictors.

## Methods

### Recruitment of study participants

Two strategies were used for data collection. Firstly, breast cancer patients that reached 6 or 12 months follow-up after surgery and their caregivers were contacted by post including the study’s background and aim. Breast cancer patients were requested to discuss study participation with their caregiver and to eventually make a joint decision. If caregivers were willing to be enrolled, an additional recruitment letter was sent to them. After the informed consent form was signed by both caregiver and researcher, participants received a brief explanation and hyperlink to the questionnaire by email. Two reminders were sent to participants who failed to complete the questionnaire after 2 and 4 weeks. Participants who did not complete the questionnaire were not included in the analysis. Secondly, the CarerQol has been disseminated through the Erasmus University Medical Center’s electronic patient reported outcome measurement (PROM) collection tool (“Zorgmonitor”) in late 2019, as part of standard care for newly diagnosed breast cancer patients and their caregivers [13]. Data from these completed CarerQoL questionnaires at 6 or 12 months postoperatively were also used for analyses. The 6 or 12 months follow up moments for completion of the CarerQoL were already determined in the PROMs collection tool prior to the concept of this study. Therefore, only breast cancer patients at 6 or 12 months post-surgery were approached during the active recruitment to maintain consistency in time since treatment.

### Data collection

CarerQoL data were prospectively collected from August 2019 to February 2021 and stored in a “LimeSurvey” database, a secure online survey tool provider [14]. Characteristics of breast cancer patients were retrospectively collected, including age and type of breast surgery. Neo-adjuvant or adjuvant systemic therapy, endocrine therapy and radiotherapy were collected as a dichotomous outcome (yes/no).

### Outcome measurements

#### CarerQoL

The CarerQoL, developed in 2006 [15], is a caregiver reported measure combining a description of the caregiving situation (CarerQol-7D) with a valuation of informal care in terms of quality of life (CarerQol VAS, a visual analogue scale for general happiness). The current study used a Dutch translation of the first version (2006). The translation was performed by the institute for Medical Technology Assessment prior to the conception and design of this study [16].

The CarerQol-7D comprises seven burden dimensions, of which 5 negative and 2 positive, each with 3 possible answer options. This includes (± indicating positive/negative dimension) fulfillment of care giving ( +), relational problems ( −), mental health problems ( −), problems with combining daily activities ( −), financial problems ( −), social support ( +) and physical health problems ( −).Answers on the negative dimensions of the CarerQol-7D receive a value of 0 (a lot), 1 (some) or 2 (no); answers on the positive dimensions receive a value of 0 (no), 1 (some), or 2 (a lot). After summing the values for the seven dimensions, the overall sum score indicates the impact of informal care on caregivers. The higher the score (range 0–14), the better the caregiver experiences providing informal care. The CarerQol utility score is a weighted sum score using utility tariffs, based on preferences of the general public for the different caregiving situations. Dutch tariffs have been published [17]. The CarerQoL VAS score ranges from 0 (worst experience of the caregiver about the informal care situation) to 10 (best experience of the caregiver about the informal care situation). The psychometric properties of the CarerQol have been investigated in previous studies. The CarerQoL demonstrated no floor or ceiling effects, with high feasibility and a reasonable degree of internal consistency in a study that used data of informal carers in Australia [18]. The CarerQoL has been validated in a large heterogeneous cohort of caregivers in the Netherlands, but not for breast cancer caregivers specifically [19–21]. Other validation studies were performed in caregivers of patients with dementia, caregivers of patients in a palliative setting, and in a large cohort of informal caregivers of older persons [22–24].

#### Health-related quality of life in breast cancer patients

HRQoL of breast cancer patients was measured with the cancer specific European Organization for Research and Treatment of Cancer Quality of Life Questionnaire (EORTC QLQ-C30) and the EQ-5D-5L [25, 26]. The EORTC QLQ-C30 is a 30-item questionnaire composed of a global quality of life (QoL) subscale, functional subscales and cancer-related symptom scales. Responses to all items were converted to a 0–100 scale. For functional and global QoL scales, higher scores represent a better level of functioning/QoL than lower scores; for symptom-oriented scales, higher scores represent greater symptom severity [25]. The score for global health status was used to compare HRQoL scores in this study. The EORTC QLQ-C30 is validated for oncology clinical research [27]. It has also been validated and found to be responsive in breast cancer patients and therefore commonly used in breast cancer research investigating HRQoL [25, 28, 29].

The EQ-5D-5L is a standardized, non-disease specific instrument to describe the HRQoL using five dimensions (mobility, self-care, usual activities, pain/discomfort, and anxiety/depression), each with five levels of functioning, ranging from no problems to extreme problems [26]. A quality-adjustment weight or “utility” is a number anchored at 0 and 1, with “perfect health” carrying a weight of 1 and death carrying a weight of 0. In this study, the pre-defined EQ-5D-5L value set of the Netherlands was used to compute utility scores based on a specific health state as indicated by a respondent [30]. The EQ VAS score is related to the EQ-5D-5L and used to rate the overall health on a scale from 0 (worst imaginable health state) to 100 (best imaginable health state) [26]. The EQ-5D-5L is widely recognized as a HRQoL measurement tool for cancer patients and has been validated in breast cancer patients [31, 32].

### Statistical analysis

Descriptive statistics, including frequencies and proportions were used to describe patient and caregiver characteristics. Medians and inter-quartile ranges (IQR) were used to present the results of the overall CarerQoL sum scores. All scores were tested for normality with the Kolmogorov–Smirnov and Shapiro–Wilk test. Scores were not normally distributed and natural log transformation was applied to all CarerQoL, EORTC QLQ-C30 and EQ-5D-5L scores. The distribution of responses to the CarerQoL-7D were calculated in percentages for each of the seven dimensions, and for 6 and 12 months post-surgery separately. Univariate and multivariable linear regression analysis were used to assess the association between kind of relationship with patient, patient’s age, type of surgery, adjuvant breast cancer treatments and log transformed CarerQoL scores. Because of the high number of male caregivers and the missing values of caregivers’ age in most cases, these variables were not included in the regression models. The multivariable linear regression analysis was stratified for time since treatment (6 and 12 months). The effect of the predictors was expressed as beta’s and the total amount of variance explained by the models in R^2^. The Spearman’s rho was used to describe the correlation of caregivers scores and the EQ VAS and EORTC QLQ-C30 scores of their respective breast cancer patient. The interpretation of the Spearman’s correlation coefficients was based on the following standards: 0.1–0.19 (very weak), 0.2–0.39 (weak), 0.4–0.59 (moderate), 0.6–0.79 (strong), and 0.8–1 (very strong) [33]. Two-sided *p*-values < 0.05 were considered statistically significant. Statistical analyses were performed using SPSS, Version 25.0 (IBM Corporation, Armonk, NY, USA) and R, Version 1.2.

### Ethical considerations

Formal approval from the local Medical Ethics Review Committee was waived as the Dutch Medical Research (Human Subjects) Act did not apply to this study.

## Results

### Study participants

From July till September 2020, a total of 153 breast cancer patients with their respective caregivers received an invitation to participate in the study. Eventually 34 caregivers responded and signed informed consent, of which two caregivers did not complete the questionnaire after sending two reminders, resulting in a response rate of 22%. Additionally, 84 completed CarerQoL questionnaires were identified in the electronic PROMs collection tool (“Zorgmonitor”). Thus, a total of 116 CarerQoL questionnaires from 2019 to 2020 were analyzed; 67 caregivers in the six months and 49 caregivers in the twelve months post-surgery group. A total of 32 caregivers completed the CarerQoL at both follow-up moments.

The majority of caregivers were male (81.4%) and the median age was 60.5 (IQR 25.0) (Table 1a). Most participants were the care recipient’s spouse or partner with a family consisting either of a partner alone or a partner and children. Median age of breast cancer patients was 54.0 (IQR 25.0) and 39.7% received chemotherapy, either neo-adjuvant or adjuvant, 58.6% received radiotherapy and 46.6% were treated with endocrine therapy (Table 1b). None of the patients had metastatic disease.

**Table 1:** a and b. Characteristics of caregivers and breast cancer patients (N = 116), 2019–2021

(a)	
*Informal caregivers (N = 116)*	
	*Median (IQR)* *N (%)*
*Age*	60.5 (25.0)
Missing (n = 68)	
*Gender*	
Male	94 (81.0)
Female	18 (15.5)
Unknown	4 (3.4)
*Relation with breast cancer patient*	
Spouse/partner	96 (82.7)
Other (parent, child, friend)	20 (15.4)
*Family status*	
Alone	8 (6.8)
Partner	40 (33.9)
Child(ren)	2 (1.7)
Partner and child(ren)	58 (49.2)
Unknown	10 (8.5)

(b)	
*Breast cancer patient or “care recipient” (N = 116)*	
	*Median (IQR)* *N (%)*
*Age*	54.0 (25)
*Type of breast surgery*	
Lumpectomy	51 (44.1)
Mastectomy	41 (35.3)
Reconstruction	24 (20.7)
Radiotherapy	68 (58.6)
Endocrine therapy	54 (46.6)
Chemotherapy	46 (39.7)
Neo adjuvant chemotherapy	31 (26.7)
Adjuvant chemotherapy	21 (18.1)

### Primary outcome

**Fig. 1:**
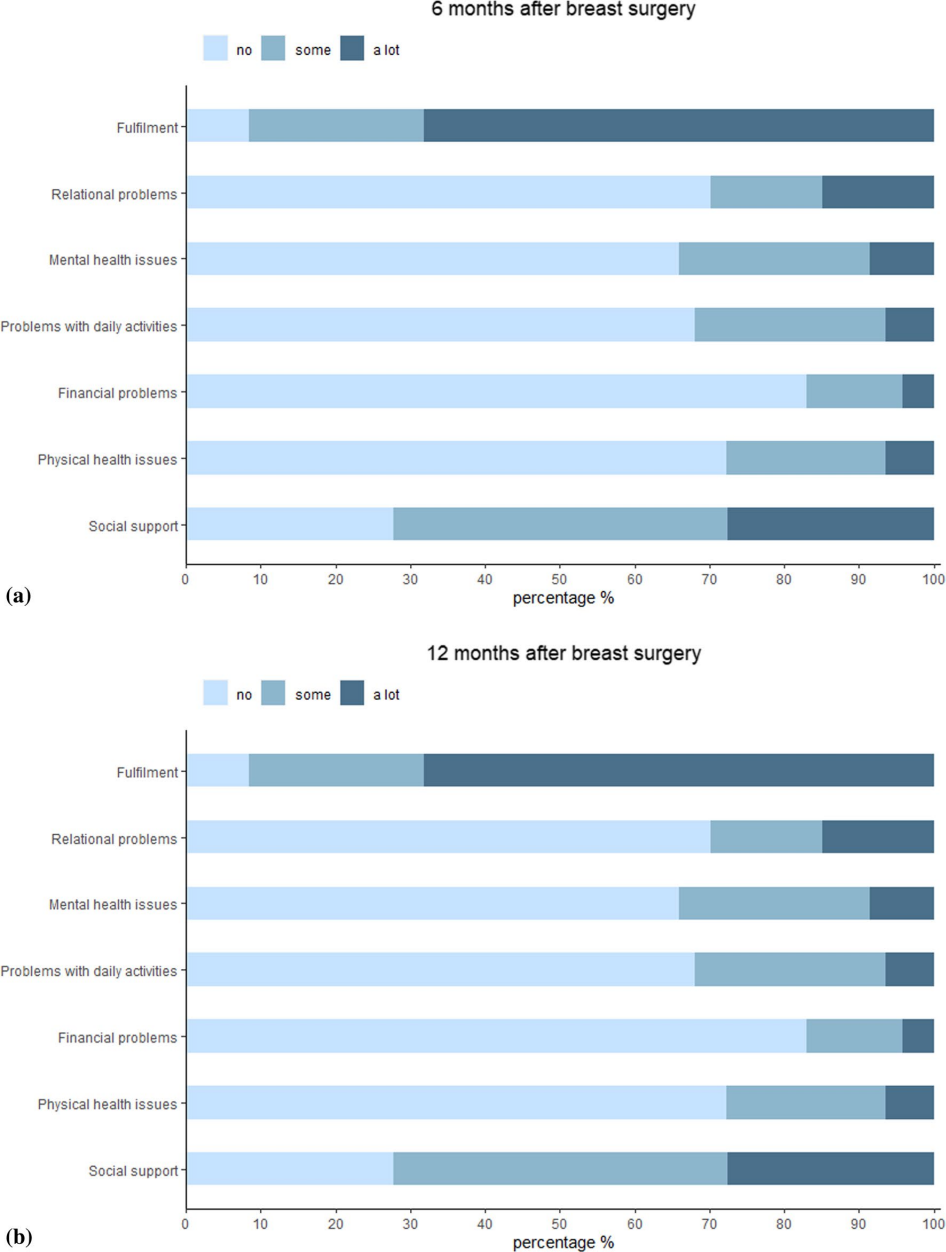
**a** and **b**. Distribution of responses to the CarerQol-7D in caregivers of breast cancer patients after 6 and 12 months follow-up (n = 67 and n = 49, respectively)

After applying the Dutch set of tariffs to each dimension of the CarerQoL-7D, the median utility score was 92.4/100 (IQR 14.9). According to the positive dimensions, most caregivers experienced some or a lot of fulfillment (98.5% vs. 91.8%) and support when needed (85.1% vs. 71.4%) at six versus twelve months (Fig. 1a and b). Median score for the CarerQol VAS score was 8.0/10 (IQR 1.0) (Table 2).

**Table 2:** Median CarerQoL, EQ-5D-5L and EORTC QLQ-C30 scores (N = 116)

	*Median (IQR)*	6 months follow-up (N = 67)	12 months follow-up (N = 49)
*Informal caregivers*			
CarerQoL Utility Score	92.4 (14.9)	92.3 (14.7)	93.6 (16.6)
CarerQoL VAS Score	8.0 (1.0)	7.0 (1.0)	8.0 (1.0)
*Breast cancer patients or “care recipient”*			
EQ-5D-5L Utility Score	0.835 (0.137)	0.818 (0.144)	0.883 (0.156)
EQ VAS Score	80 (25)	80 (20)	80 (26)
EORTC QLQ-C30 Global health status	83.3 (25)	75 (16.7)	83.3 (16.7)

CarerQoL = Carer Quality of Life, VAS = Visual Analogue Scale, IQR = interquartile range

### Correlations between caregivers’ and breast cancer patients’ quality of life scores

The CarerQoL VAS score was positively but weakly correlated with the patients’ EQ-5D-5L utility score (0.301, *p* = 0.002), EQ VAS score (0.251, *p* = 0.009) and EORTC QLQ-C30 global health status (0.339, *p* < 0.001). The CarerQoL utility score showed a weak correlation with patient’s EORTC QLQ-C30 global health status (0.236, *p* = 0.015). See Table 3.

**Table 3:** Spearman’s correlation coefficients (N = 116)

	CarerQol VAS Score	CarerQoL utility score	EQ VAS score	EQ-5D-5L utility score	EORTC QLQ-C30 Global health status
CarerQoL VAS Score		0.520 (*p* < 0.001)*	0.251 (*p* = 0.009)*	0.301 (*p* = 0.002)*	0.339 (*p* < 0.001)*
CarerQoL utility Score	0.520 (*p* < 0.001)*		0.023 (*p* = 0.810)	0.148 (*p* = 0.126)	0.236 (*p* = 0.015)*
EQ VAS score	0.251 (*p* = 0.009)*	0.023 (*p* = 0.810)		0.678 (*p* < 0.001)*	0.608 (*p* < 0.001)*
EQ-5D-5L Utility score	0.301 (*p* = 0.002)*	0.148 (*p* = 0.126)	0.678 (*p* < 0.001)*		0.556 (*p* < 0.001)*

CarerQoL = Carer Quality of Life, VAS = Visual Analogue Scale. EQ-5D-5L utility and EQ VAS scores and EORTC QLQ-C30 Global health status of breast cancer patients

*Correlation is significant at the level 0.05 (two-tailed)

### Univariable and multivariable regression analyses

Data of 116 caregivers at six and twelve months follow up was included in the univariable regression analysis, in which four caregiver characteristics and five patient characteristics were analyzed. Relationship between caregiver and breast cancer patient (partner/spouse versus other) was positively related with caregivers’ log transformed CarerQoL utility score (B = 0.106, *p* = 0.034). Chemotherapy was associated with the CarerQoL utility score (B =  − 0.097, *p* = 0.019) and to a lesser extent with the CarerQoL VAS score (B = 0.036, *p* = 0.125). This was also the case for the association between age of breast cancer patient and CarerQoL utility score (B = 0.001, *p* = 0.034) and CarerQoL VAS score (B = 0.002, *p* = 0.078).

The multivariable regression analysis revealed chemotherapy as a significant negative predictor for the log transformed CarerQoL utility score (B =  − 0.063, *p* = 0.001) and log transformed CarerQoL VAS score (B =  − 0.044, *p* = 0.038) at 6 months follow-up. Adjusted R^2^ for the models was 0.188 and 0.165 respectively. At twelve months follow up, results for the log transformed CarerQoL utility score were B =  − 0.010 (*p* = 0.758, Adjusted R^2^ = 0.126) and B =  − 0.042 (i = 0.601, Adjusted R^2^ =  − 0.121) for the CarerQoL VAS score (Table 4).

**Table 4:** a and b. Multivariable linear regression coefficients for the log-transformed CarerQoL utility and VAS score after 6 and 12 months follow-up

	CarerQoL utility score			CarerQoL VAS score		
	Beta	Std. error	Sig	Beta	Std. error	Sig
(a) T = 6 months						
Age breast cancer patient	− 7.289E−5	0.001	0.924	0.001	0.001	0.092
Relationship between caregiver and breast cancer patient	0.020	0.018	0.278	-0.008	0.021	0.718
Chemotherapy	**− 0.063**	**0.018**	0.001*	**− 0.044**	**0.020**	0.038*
Radiotherapy	0.036	0.030	0.242	0.058	0.035	0.1
Adjuvant endocrine therapy	− 0.002	0.017	0.920	0.019	0.019	0.334
*Type of surgery*						
Lumpectomy	Ref			Ref		
Mastectomy	0.044	0.026	0.091	0.028	0.030	0.352
Reconstruction	0.035	0.037	0.341	0.039	0.042	0.360

(b) T = 12 months						
Age breast cancer patient	− 3.433E−5	0.001	0.977	− 0.001	0.003	0.778
Relationship between caregiver and breast cancer patient	− 0.002	0.028	0.954	− 0.031	0.070	0.656
Chemotherapy	− 0.010	0.031	0.758	− 0.042	0.079	0.601
Radiotherapy	− 0.061	0.051	0.237	− 0.091	0.128	0.484
Adjuvant endocrine therapy	− 0.009	0.028	0.735	0.018	0.070	0.797
*Type of surgery*						
Lumpectomy	Ref			Ref		
Mastectomy	− 0.032	0.046	0.701	− 0.011	0.115	0.925
Reconstruction	− 0.033	0.062	0.591	− 0.117	0.155	0.455

*Significance at the level 0.05 (two-tailed)

## Discussion

While much effort is generally expended on providing social support for breast cancer patients during treatment, little attention has been paid to the needs of the caregiver in daily practice. The primary aim of this study was to evaluate the quality of life of caregivers of breast cancer patients using the CarerQoL utility and VAS score. In addition, the association with breast cancer patients’ HRQoL and potential predictors of the caregivers’ quality of life were evaluated.

The results of this survey indicates that the overall care-related quality of life of caregivers is good, based on a median CarerQoL utility score of 87.0/100 and VAS score of 8.0/10. The caregivers of breast cancer patients in our cohort formed a homogeneous group, as most caregivers were male spouses or partners. The role of caregiving may be experienced differently between spouses and non-spouses (e.g. close friends or relatives) [35]. Worldwide, caregivers of cancer patients are most often females, experiencing higher levels of caregiving burden. On the contrary, levels of distress may be determined by gender, with females having higher distress levels regardless of their role (e.g. patient or caregiver) [36]. Our results suggest that caregiving does not completely disrupt caregivers’ lives and relationships with breast cancer patients, but could affect the seven dimensions of the social environment in some cases. The question rises to what extent changes in scores are considered to be clinically meaningful. Minimal clinically important differences (MCID) indicate the smallest change in PROM scores which subjects perceive to be important or beneficial, and which would justify an intervention or change in management [37]. MCIDs for the CarerQoL or measures to determine MCIDs for caregivers have not been described previously. The CarerQoL was initially developed for economic evaluations of healthcare, as health care interventions impact both patients and the caregiver burden. When the total societal perspective is evaluated in such cost-effectiveness studies, the optimal approach would be to also include informal care outcomes. However, this may be the most universal questionnaire to evaluate the care-related quality of life in caregivers of cancer patients.

One of the strengths of this study is that the caregivers’ CarerQol scores were correlated to HRQoL scores of breast cancer patients. A positive but weak to moderate correlation was observed between the CarerQoL scores of caregivers and the HRQoL scores of breast cancer patients. It was assumed that care-related quality of life scores of caregivers could reflect the HRQoL of breast cancer patients and vice versa. In caregiving for other diseases, such as Alzheimer or Parkinson’s disease, the caregiver burden was inversely associated with the quality of life of patients [38, 39]. However, our results suggest that although the HRQoL of breast cancer patients diminishes over time, this does not directly impact the care-related quality of life of caregivers. Such observations can possibly be used to manage the caregivers’ expectations prior to treatment.

Another strength is using the CarerQoL Instrument to evaluate the care-related quality of life in caregivers of breast cancer patients, as this has never been described in the literature before. Translating the results of previous caregiver-related studies into daily practice remains challenging. For example, outcomes based on a review of Lopes et al. are only useful to a certain extent as objective measurements are lacking [35]. In three other studies, the psychosocial impact of caregiving in women with advanced breast cancer in a palliative setting or recurrent disease was described [40–42]. Overall, they conclude that patient’s physical and emotional factors can predict the caregivers’ quality of life. According to the Short Form 36 questionnaire, better quality of life scores in patients and caregivers were found if the caregiver was spouse. Formal comparisons with our results was difficult, as this current cohort did not include metastasized or recurrent breast cancer and one study did not use validated questionnaires. The breast cancer patients that were linked to the caregivers in this study made up a small and heterogeneous group according to the treatment characteristics. Treatment strategies for breast cancers have different symptom burden and duration of therapy, which may have an impact on the intensity of care provided by caregivers. This may also influence the tasks and medical support carried out by caregivers. Our results suggest that chemotherapy in breast cancer patients was negatively associated with quality of life scores of caregivers, but previous research found inconsistent results [43–45]. According to a study of Nijboer et al., numerous background characteristics of the caregiver may influence the quality of life, including age, gender, living situation, socioeconomic status and type of relationship between care recipient and caregiver [46]. Another study investigated potential determinants that influence the quality of life of Chinese caregivers of specifically breast cancer patients. Quality of life was measured with the Short Form-36 questionnaire. Although they found several significant associated predictors (income, educational level and symptom severity) which were unfortunately not included in our analyses, similar non-significant correlations for overlapping variables were found [43]. As chemotherapy may contribute to symptom severity, this may explain the negative association with our CarerQoL scores at six months follow up and that the effect on quality of life is normalized after one year. The relationship with breast cancer patients was not investigated, as only spouses were included in their study.

## Study limitations

Several limitations were identified in this study. Firstly, although several reminders were sent to participants to complete the questionnaire, one third responded. The low response rate may be due to the fact that caregivers did not directly identify themselves as such. As previously mentioned, caregivers often experience the provision of informal care as an obligation to their family, and something that goes without saying. This may have resulted in some selection bias. On the other side, by disseminating the survey directly to caregivers, we maybe have reached more persons lending informal care who would normally not define themselves as caregivers, for instance because their burden is low. The suboptimal response rate could have influenced the normal distribution of CarerQoL scores, for which log transformation was applied. It is possible that the caregivers with a low burden and relatively good quality of life may be more likely to complete a survey. However, caregivers that have a higher burden may be more self-conscious of their care-related problems, recognize the importance of such research, and are willing to participate in a survey.

Secondly, the lack of socio-demographic characteristics of caregivers prevents to precisely describe the cohort that was studied. Educational level and current work situation can be important explanatory factors of the care-related quality of life. Such variables were only registered for those caregivers that completed the questionnaire after active recruitment and not during standard breast cancer care. Due to the low response rate, these data were not sufficient enough to use in the analysis.

Lastly, the questionnaires were only administered to caregivers of patients of a tertiary hospital with a specialized academic breast cancer center in which more younger women or advanced stages of breast cancer are treated. Therefore, the results may not be generalizable to patients of general hospitals.

## Clinical implications

Results highlight the need to include caregivers of breast cancer patients in clinical practice, and provide reference values in a predefined cohort of caregivers of breast cancer patients. The CarerQoL has already been implemented in the institutes electronic PROM collection tool since 2019. Providing feedback and discussing questionnaire outcomes is important to maintain adherence and to act upon if quality of life is diminishing. However, physicians usually do not have a doctor-patient relationship with the caregiver. An implication for clinical practice could be exchanging the CarerQoL outcomes with general practitioners. They could evaluate the quality of life scores of caregivers but also provide additional support or care for the caregiver if needed. In addition, caregivers could benefit from self-management tasks in managing their own quality of life. The CarerQoL may be a suitable tool in optimizing self-management to prevent caregiver-related health issues.

## Conclusions

This is the first study that evaluated the CarerQoL Instrument in caregivers of breast cancer patients. Most caregivers felt happy as they were satisfied and experienced fulfillment in their role as caregiver. A minority of the caregivers indicated some problems in their relationship, mental and physical health, finances, or daily activities. Caregivers’ happiness was associated with breast cancer patients’ HRQoL. Chemotherapy was a negative predictor for logtransformed CarerQoL utility and VAS scores as six months follow-up. Results of this study can be used as a reference for future quality of life evaluations in caregivers of breast cancer patients.

## Data Availability

Data shall only be shared with researchers upon reasonably request, at the discretion of the principal investigator.
