# Correction: Quality of life of caregivers of breast cancer patients: a cross-sectional evaluation

**DOI:** 10.1186/s12955-022-02028-3

**Published:** 2022-07-26

**Authors:** Marloes E. Clarijs, Arvind Oemrawsingh, Mirelle E. E. Bröker, Cornelis Verhoef, Hester Lingsma, Linetta B. Koppert

**Affiliations:** 1 Academic Breast Cancer Center, Department of Surgical Oncology and Gastrointestinal Surgery, Erasmus MC Cancer Institute, Erasmus University Medical Center, Room NA-2117, 3000 WB Rotterdam, The Netherlands; 2 Center for Medical Decision Making, Department of Public Health, Erasmus University Medical Center, Rotterdam, The Netherlands

## Correction to: Health and Quality of Life Outcomes (2022) 20:29 10.1186/s12955-022-01930-0

The original article contained a duplicated sub-figure in place of Fig. 1a. The correct sub-figure has since been re-implemented.

